# Dissecting the interaction of photosynthetic electron transfer with mitochondrial signalling and hypoxic response in the Arabidopsis *rcd1* mutant

**DOI:** 10.1098/rstb.2019.0413

**Published:** 2020-05-04

**Authors:** Alexey Shapiguzov, Lauri Nikkanen, Duncan Fitzpatrick, Julia P. Vainonen, Richard Gossens, Saleh Alseekh, Fayezeh Aarabi, Arjun Tiwari, Olga Blokhina, Klára Panzarová, Zuzana Benedikty, Esa Tyystjärvi, Alisdair R. Fernie, Martin Trtílek, Eva-Mari Aro, Eevi Rintamäki, Jaakko Kangasjärvi

**Affiliations:** 1 Organismal and Evolutionary Biology Research Programme, Faculty of Biological and Environmental Sciences, University of Helsinki, FI-00014 Helsinki, Finland; 2 Viikki Plant Science Center, University of Helsinki, FI-00014 Helsinki, Finland; 3 Department of Biochemistry/Molecular Plant Biology, University of Turku, FI-20014 Turku, Finland; 4 Max-Planck Institute for Molecular Plant Physiology, D-14476 Potsdam-Golm, Germany; 5 Center of Plant Systems Biology and Biotechnology, 4000 Plovdiv, Bulgaria; 6 Photon Systems Instruments, 664 24 Drásov, Czech Republic

**Keywords:** *Arabidopsis thaliana*, photosynthetic electron transfer, reactive oxygen species, hypoxia, mitochondrial dysfunction stimulon, retrograde signalling

## Abstract

The Arabidopsis mutant *rcd1* is tolerant to methyl viologen (MV). MV enhances the Mehler reaction, i.e. electron transfer from Photosystem I (PSI) to O_2_, generating reactive oxygen species (ROS) in the chloroplast. To study the MV tolerance of *rcd1*, we first addressed chloroplast thiol redox enzymes potentially implicated in ROS scavenging. NADPH-thioredoxin oxidoreductase type C (NTRC) was more reduced in *rcd1*. NTRC contributed to the photosynthetic and metabolic phenotypes of *rcd1*, but did not determine its MV tolerance. We next tested *rcd1* for alterations in the Mehler reaction. In *rcd1*, but not in the wild type, the PSI-to-MV electron transfer was abolished by hypoxic atmosphere. A characteristic feature of *rcd1* is constitutive expression of mitochondrial dysfunction stimulon (MDS) genes that affect mitochondrial respiration. Similarly to *rcd1*, in other MDS-overexpressing plants hypoxia also inhibited the PSI-to-MV electron transfer. One possible explanation is that the MDS gene products may affect the Mehler reaction by altering the availability of O_2_. In green tissues, this putative effect is masked by photosynthetic O_2_ evolution. However, O_2_ evolution was rapidly suppressed in MV-treated plants. Transcriptomic meta-analysis indicated that MDS gene expression is linked to hypoxic response not only under MV, but also in standard growth conditions.

This article is part of the theme issue ‘Retrograde signalling from endosymbiotic organelles’.

## 1. Introduction

Perturbations of mitochondrial electron transfer trigger retrograde signalling that activates the expression of the mitochondrial dysfunction stimulon (MDS) genes in the nucleus [1–3]. In Arabidopsis, the MDS signalling pathway is regulated by at least two transcription factors, ANAC013 [1] and ANAC017 [4], and is inhibited by the nuclear co-regulator protein RCD1 [3]. As expected, most proteins encoded by the MDS genes are related to mitochondrial functions. However, the studies in plants with enhanced MDS gene expression, including the *rcd1* mutant [3,5–7], the *ANAC013* overexpressor [1] and the *ANAC017* overexpressor [2], have also revealed alterations in their chloroplasts. The characteristic feature of the MDS-overexpressing lines is their tolerance to methyl viologen (MV, also known as paraquat) [1–3]. MV catalyses electron transfer from Photosystem I (PSI) to molecular oxygen (O_2_), referred to as the Mehler reaction [8,9]. MV promotes oxidation of PSI, modifies redox state of the photosynthetic electron transfer (PET) chain and allows the Mehler reaction to outcompete other electron fluxes downstream from PSI, including cyclic electron transfer (CET) [3,10–12]. The Mehler reaction is the main source of reactive oxygen species (ROS) in illuminated chloroplasts. Accordingly, in MV-treated plants, gradual light-dependent increase in ROS production rate ultimately leads to destabilization of Photosystem II (PSII) and to cell death [3,13,14].

How the MDS gene products provide resistance to MV is unknown. One possible component of the resistance is altered redox state of chloroplast thiol enzymes. The *rcd1* mutant was isolated in several genetic screens related to altered tolerance to ROS [15–17]. In addition, it has been identified in a genetic screen for chloroplast redox imbalance [5,6]. Recently, we discovered that the pool of the abundant chloroplast 2-Cys peroxiredoxin (2-CP) is more reduced in *rcd1* than in the wild type [3]. This could contribute to MV tolerance, as the ability of 2-CP to scavenge ROS is well established [18–21]. Another possible basis for MV tolerance of *rcd1* is alterations of electron transfer downstream of PSI. These alterations have been associated with the activity of mitochondrial alternative oxidases (AOXs) [3]. AOXs are encoded by MDS genes. These mitochondrial enzymes with ubiquinol:oxygen oxidoreductase activity provide an extra-chloroplastic electron sink for PET [22–25]. Pharmacological or genetic inhibition of AOX activity suppresses photosynthesis [24,26], modifies PET and decreases the tolerance of plants to MV [3], but the mechanisms remain unknown.

Here, we studied the response of wild type, *rcd1* and other MDS-overexpressing plants to MV to gain insight into the mechanisms whereby MDS gene products affect the chloroplast. Our results suggest that in the plants with enhanced MDS expression, the electron transfer through MV was inhibited by hypoxic environment. One of the possible explanations for this is that the interaction between the organelles may be linked to alterations in cellular oxygen availability.

## 2. Material and methods

### (a) Plant lines and growth conditions

*Arabidopsis thaliana* (Col-0) plants were cultivated on soil (1 : 1 peat:vermiculite) at a 12 h photoperiod and light intensity of 220–250 µmol m^−2^ s^−1^. For measuring light-harvesting antenna complex II (LHCII) phosphorylation, seedlings were grown for 12 days on MS basal medium (Sigma-Aldrich) with 0.5% Phytagel (Sigma-Aldrich) without sucrose, at a 12 h photoperiod and light intensity of 150–180 µmol m^−2^ s^−1^. Arabidopsis *rcd1–4* (GK-229D11), *ntrc* (SALK 096776), *npq4–1* [27], *ptox* [28], *stn7* (SALK 073254), *aox1a* (SAIL 030_D08), *aox1c* (SAIL 96_D08) and *aox1d* (GK-529D11) mutants, *NTRC* overexpressor [29], *ANAC013* overexpressor [1] and *AOX1a* overexpressor [30] lines are of Col-0 background. The *rcd1 aox1a* double mutant has been described in [31].

### (b) Chemical and hypoxic treatments

Leaf discs were placed on Milli-Q water with 0.05% Tween-20 (Sigma-Aldrich) +/− MV. Unless specified otherwise, 1 µM MV was used. Final concentration of antimycin A (AA) was 2.5 µM [3]. Dark pre-treatment with MV and AA was overnight. To generate hypoxic atmosphere, nitrogen gas was flushed inside a custom-built chamber containing plant material. Imaging was performed through the glass cover. Alternatively, plant material was placed into the AnaeroGen Compact anaerobic gas generator bag (Oxoid). AnaeroGen decreases oxygen concentration below 0.5% producing 9–13% CO_2_ [32]. CO_2_ accumulation was prevented by LoFloSorb non-caustic containing carbon dioxide absorbent (Intersurgical). O_2_ was controlled with resazurin anaerobic indicator (Oxoid).

### (c) Thiol-specific labelling of protein extracts

Thiol-specific labelling of protein extracts was done and interpreted as described in [3].

### (d) Feeding with ^14^C glucose and analysis of metabolic fluxes

^14^C glucose labelling, fractionation and analysis of metabolic fluxes were performed as described in [3]. Arabidopsis leaf discs were incubated for 150 min in light with 5 ml of 10 mM MES-KOH (pH 6.5) containing 1.85 MBq mmol^−1^ [U-^14^C] glucose (Hartmann Analytic) in a final concentration of 2 mM. Leaf discs of the dark experiment were incubated similarly, but under the green light. Samples were washed with distilled water, harvested and kept at −80°C for further analysis. The evolved ^14^CO_2_ was collected in 0.5 ml of 10% (w/v) KOH. Samples were extracted, fractioned and metabolic fluxes were analysed according to [3]. Material from frozen leaf discs was extracted in a two-step ethanolic extraction of 80% (v/v) and 50% (v/v). Supernatants were combined, dried and resuspended in 1 ml of water [33,34]. The soluble fractions were separated into neutral, anionic and basic fractions by ion-exchange chromatography as described in [34]. About 2.5 ml of the neutral fraction were freeze-dried and resuspended in 100 µl of water for further enzymatic digestions as described in [35]. Phosphate esters of the soluble fractions were measured as in [3] and starch of the insoluble fractions was measured as described in [33]. Calculation of the fluxes was performed according to the assumptions described by Geigenberger *et al*. [36,37].

### (e) Pulse-amplitude-modulation chlorophyll fluorescence imaging

Measurements of chlorophyll fluorescence kinetics and long-term PSII inhibition were performed using Walz Imaging pulse-amplitude-modulation (PAM) fluorometer as described in [3].

### (f) Measurement of proton motive force by electrochromic shift

Measurement of proton motive force was done as described in [38].

### (g) Measurement of gas exchange by membrane inlet mass spectrometry

Fourteen-mm leaf discs were floated overnight in Milli-Q water with Tween-20 ±1 µM MV at 20°C. Following the overnight incubation, 12.5 mm discs were cut from the centre of the pre-treated discs in very dim light and loaded into a sealed membrane inlet mass spectrometry (MIMS) cuvette calibrated to 22°C. The cuvette was purged using air scrubbed of ^12^CO_2_ with carbosorb before ^13^CO_2_ gas (99% ^13^CO_2_, Sigma-Aldrich) was injected to approximately 2% by volume, and ^18^O_2_ gas (98% ^18^O_2_, Cambridge Isotope Laboratories, UK) was enriched to approximately 3%. Samples were kept in darkness until gasses equilibrated between all areas of the leaf (approximately 5 min). Then data acquisition was commenced, comprising 3 min darkness, 7 min light (120 µmol m^−2^ s^−1^) and 3 min darkness. Light was provided by a halogen bulb *via* a liquid light guide. Masses 32, 36, 44 and 45 were monitored with a Sentinel Pro magnetic sector mass spectrometer (Thermo Fisher, USA) allowing the calculation of O_2_ evolution by PSII (mass 32), O_2_ consumption by terminal oxidases and Mehler-type pathways (mass 36), CO_2_ production by mitochondrial activity [minus internal CO_2_ recaptured (mass 44)] and CO_2_ fixation by Rubisco [minus internal CO_2_ recaptured (mass 45)]. Data processing was based on concepts and methods described by [39].

### (h) Direct fast imaging of OJIP chlorophyll fluorescence kinetics

The imaging of OJIP (Fo, Fj, Fi, Fp) fluorescence kinetics was performed using FluorCam FC800F from Photon Systems Instruments, Czech Republic (www.psi.cz). The instrument is described in [40]. It contains an ultra-fast sensitive CMOS camera, TOMI 3, developed by Photon Systems Instruments, which performs image acquisition with maximum frame rate of 20 µs. FluorCam software was used to control the instrument and to analyse the data. The OJIP imaging protocol involved the triple measurement of the background signal followed by three 20 µs flashes of saturating light to measure Fo and then a 1 s saturating light pulse (3 500 µmol m^−2^ s^−1^) to follow OJIP kinetics. Both the background and the Fo values were averaged for further calculations. The frame period was set at 300 µs, and the integration time was 35–50 µs. The excitation light was generated by a pair of blue LED panels (470 nm) and filtered by dichroic filters that block light at 490–800 nm to avoid crosstalk with the detection. To record chlorophyll fluorescence signal, the camera was equipped with 700- to 750-nm band filters.

### (i) Transcriptomic meta-analyses

Gene expression data were acquired from ArrayExpress E-MTAB-662 (*rcd1*) [31], E-GEOD36011 (3 h antimycin A) [41] and E-GEOD-9719 (2 h hypoxia) [42,43]. Genes that both showed at least a 2-fold change and had a statistical significance of *p* < 0.05 were considered as differentially expressed and were categorized as up- or down-regulated based on the direction of the change. The overlap of multiple gene lists was analysed using Venn diagrams. Pairwise Fischer's exact test was performed on the gene lists.

## 3. Results

### (a) NADPH-thioredoxin oxidoreductase type C is more reduced in *rcd1*, but this does not explain methyl viologen tolerance of the mutant

**Figure 1.**
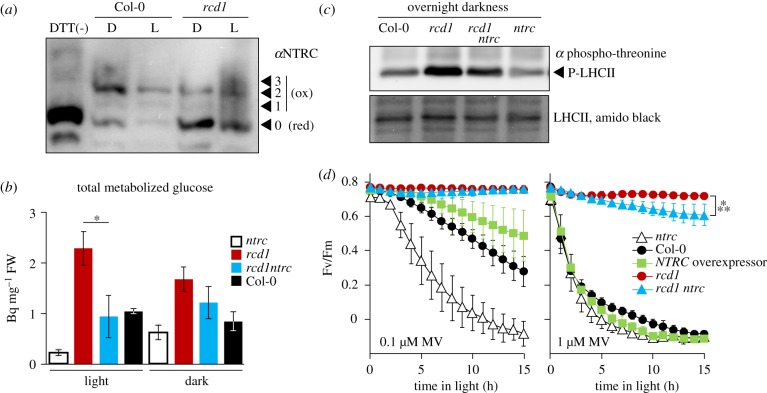
NTRC contributes to the phenotypes of *rcd1*. (*a*) Chloroplast NTRC pool is more reduced in *rcd1* both in darkness (D) and light (L). Thiol bond-specific labelling was performed as described in [3]. In brief, leaf protein extracts were treated with N-ethylmaleimide to block free thiol groups, then with DTT to reduce *in vivo* thiol bridges, and then with 5-kDa methoxypolyethylene glycol maleimide to label all the newly opened thiol groups. The samples were separated in SDS-PAGE and immunoblotted with the *α*NTRC antibody. DTT(-) control contains mainly unlabelled form. The unlabelled form (0) corresponds to *in vivo* reduced (red), while the labelled forms (1, 2, 3) to *in vivo* oxidized (ox) fractions of NTRC (*N* = 2). (*b*) Total metabolized radiolabelled ^14^C glucose treated to light- or dark-acclimated rosettes (mean values ± standard errors). *P-value = 0.023, one-way ANOVA with Bonferroni corrected *post hoc* test. The full dataset is presented in the electronic supplementary material, table S1. (*c*) Phosphorylation of LHCII in overnight dark-acclimated seedlings determined by immunoblotting with anti-phospho-threonine antibody. Lower panel: amido black staining of total LHCII. (*d*) The tolerance to MV-induced PSII inhibition in presence of 0.1 µM MV (left), or 1 µM MV (right). Mean values ± standard deviations are shown. ***P-value < 0.001, one-way ANOVA with Bonferroni corrected *post hoc* test (*N* = 3). Source data and statistics are presented in electronic supplementary material, table S2.

Previous study suggested that the tolerance the Arabidopsis mutant *rcd1* to MV could be explained by more reduced thiol redox state of the chloroplast thiol enzymes, in particular 2-CPs, and/or by alterations in the electron transfer downstream of PSI [3]. We first addressed thiol redox enzymes. The main *in vivo* reductant of chloroplast 2-CPs is NADPH-thioredoxin oxidoreductase type C (NTRC) encoded by a single gene in Arabidopsis [44–46]. We performed thiol bond-specific labelling of NTRC and found that it was more reduced *in vivo* in *rcd1* than in the wild type (Col-0), both in darkness and light (figure 1*a*). To address the role of NTRC in the phenotypes of *rcd1*, we generated an *rcd1 ntrc* double mutant. Analysis of metabolic fluxes revealed that the metabolism of glucose, significantly elevated in *rcd1* in light, was suppressed to wild-type levels in *rcd1 ntrc* (figure 1*b*; electronic supplementary material, table S1 for the full dataset). This hinted that alterations in the energy metabolism observed in *rcd1* were partially mediated by NTRC.

In addition to reducing 2-CPs, NTRC controls many chloroplast processes, including the activity of ATP synthase [47,48] and of thylakoid NADH dehydrogenase (NDH) complex that mediates CET from PSI to the plastoquinone pool [38]. Hence, we next evaluated NDH activity in *rcd1* by assessing the redox state of the plastoquinone pool in darkness. Reduced plastoquinone pool activates the chloroplast state transition kinase STN7 that phosphorylates the light-harvesting antenna complex II (LHCII). Therefore, LHCII phosphorylation may serve as an indirect measure of the plastoquinone redox state. Immunoblotting of total protein extracts from dark-acclimated seedlings with anti-phospho-threonine antibody revealed increased phosphorylation of LHCII in *rcd1* that was suppressed in *rcd1 ntrc* (figure 1*c*). This suggested that NDH activity was increased in *rcd1*, which is likely mediated by NTRC. Thus, the alterations of chloroplast electron flows in *rcd1* were partially NTRC dependent.

We next measured tolerance of *rcd1 ntrc* to MV-induced PSII inhibition. It was only slightly lower than in *rcd1* (figure 1*d*). It is noteworthy that, the single *ntrc* mutant was more sensitive, while the *NTRC* overexpressor line [29] was more tolerant to MV than Col-0 (figure 1*d*). Thus, the activity of NTRC contributed to, but was not the main reason for the MV resistance of *rcd1*. An alternative explanation for MV tolerance of *rcd1* suggests that it arises from changed electron transfer downstream of PSI [3]. To explore this possibility, we next addressed the effects of MV on PET *in planta*.

### (b) Methyl viologen induces non-photochemical quenching and suppresses photosynthetic oxygen evolution

**Figure 2.**
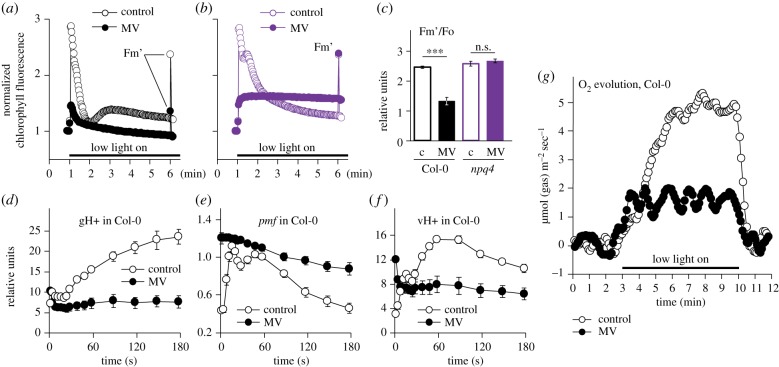
MV induces non-photochemical quenching (NPQ) and inhibits oxygen evolution in the first minutes of illumination. (*a*,*b*) Chlorophyll fluorescence was followed during 5 min of illumination with low light (80 µmol m^−2^ s^−1^) in Col-0 (*a*) and *npq4* (*b*), with or without MV. The reads are normalized to Fo. After 5 min of illumination, maximal fluorescence under light (Fm’) was measured with a saturating light flash. (*c*) Quantification of Fm’ values obtained as in (*a*,*b*). Controls untreated with MV are labelled ‘c’. ***P-value < 0.001, one-way ANOVA with Bonferroni corrected *post hoc* test. (*d*) Thylakoid proton conductivity (gH+), (*e*) proton motive force (*pmf*) and (*f*) proton flux inside the lumen (vH+) determined in Col-0 by electrochromic shift. Mean values ± standard errors are shown. (*g*) MIMS measurements of O_2_ evolution in Col-0. The full dataset is presented in the electronic supplementary material, figure S1. Source data and statistics are presented in electronic supplementary material, table S2.

To understand how MV treatment affects the performance of photosynthetic apparatus *in planta*, we first measured the kinetics of chlorophyll fluorescence using the imaging PAM. The wild-type leaves were pre-treated with the catalytic amounts of MV and then exposed to low intensity light. Importantly, the measurements were performed within the first minutes of illumination, which minimizes ROS-induced irreversible damage to PSII. MV led to pronounced decrease in maximal fluorescence under light (Fm’) as compared to the MV-untreated control (figure 2*a*). In contrast with Col-0, MV did not lower Fm’ in the *npq4* mutant that is deficient in non-photochemical quenching (NPQ; figure 2*b,c*). This suggested that the quenching effect of MV on chlorophyll fluorescence was owing to accelerated generation of NPQ.

We next aimed to explore the reasons for the MV-stimulated NPQ. NPQ is activated by light-dependent acidification of thylakoid lumen. To find out whether MV promoted lumen acidification, we assessed proton motive force (*pmf*) by measuring electrochromic shift. As expected, MV strongly inhibited thylakoid proton conductivity (gH+), resulting in elevated *pmf* and rapid decrease in proton flux from stroma into the lumen (vH+; figure 2*d–f*). Proton conductivity largely depends on the activity of thylakoid ATP synthase [49]. Taken together, these results suggested that the illumination of MV-treated plants triggered fast inhibition of thylakoid ATP synthase, which caused the rapid acidification of thylakoid lumen and increase in NPQ.

NPQ competes with charge separation in PSII, thus suppressing photosynthetic O_2_ evolution. To test whether treatment with MV indeed inhibited O_2_ evolution *in planta*, we addressed the effect of MV using MIMS. This technique allows simultaneous real-time monitoring of multiple compounds produced and absorbed through leaf gas exchange [39]. As expected, treatment with MV markedly inhibited O_2_ evolution already during the first minutes of illumination (figure 2*g*). Importantly, the concomitant MIMS measurement of CO_2_ evolution suggested that MV did not affect respiration (details in electronic supplementary material, figure S1). Thus, treatment with MV largely eliminated photosynthetic oxygen evolution but not respiration.

### (c) The effect of methyl viologen on photosynthetic electron transfer is reversibly suppressed by light in *rcd1*

**Figure 3.**
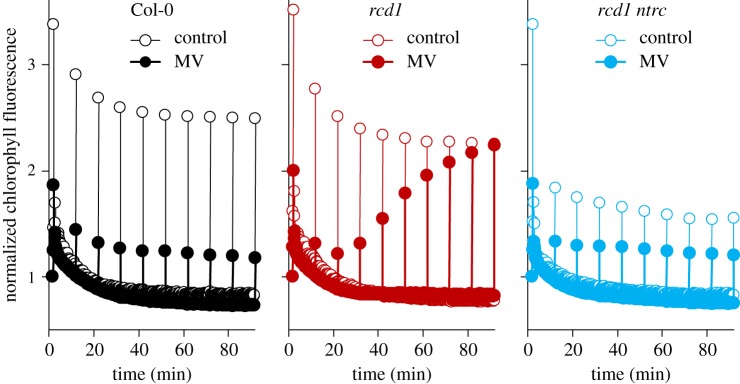
Exposure to light suppresses physiological activity of MV in the *rcd1* mutant. Kinetics of chlorophyll fluorescence during 90 min of low light (80 µmol m^−2^ s^−1^) in Col-0 (left), *rcd1* (middle) and *rcd1 ntrc* (right). Saturating light pulses were triggered once in 10 min to measure Fm’. The kinetics are normalized to Fo (*N* = 3). Source data are presented in electronic supplementary material, table S2.

We next assayed the above-described reactions to MV in the *rcd1* mutant. In MV-treated *rcd1*, the dynamics of oxygen evolution and chlorophyll fluorescence was identical to Col-0 during the first minutes of illumination (electronic supplementary material, figures S1, S2A, B). However, longer light treatment led to gradual recovery of Fm’ in *rcd1*, but not in Col-0 nor in *rcd1 ntrc* (figure 3). To find out whether the changes in Fm’ were related to release of NPQ, we generated an *rcd1 npq4* double mutant. In *rcd1 npq4*, the recovery of Fm’ was suppressed (electronic supplementary material, figure S2C). These experiments indicated that exposure to light gradually released MV-induced NPQ in *rcd1*, and this process was NTRC dependent.

To test whether in *rcd1* light promoted export of MV from its chloroplast site of action, we interrupted illumination with 20 min dark periods. After each dark treatment, NPQ restored and then gradually relaxed again (electronic supplementary material, figure S2D), making the possibility of MV export unlikely. Collectively, these observations suggested that electron flow through MV was reversibly inhibited by light in *rcd1*, but the mechanism remained unclear.

The results hinted that in illuminated *rcd1* chloroplasts, MV displays different physiological activities from in Col-0 and this is not owing to removal of MV from its site of action. MV tolerance of *rcd1* has previously been linked to altered mitochondrial respiration [3]. Both mitochondrial respiration and the electron flow through MV depend on molecular oxygen. MV inhibited photosynthetic O_2_ evolution, but not respiration (figure 2*g*; electronic supplementary material, figure S1). This raised the question whether MV treatment of *rcd1* could affect the availability of O_2_ inside the leaf, prompting us to study the effects of MV in hypoxic environment.

### (d) The methyl viologen-dependent response in *rcd1* is eliminated in hypoxic environment

**Figure 4.**
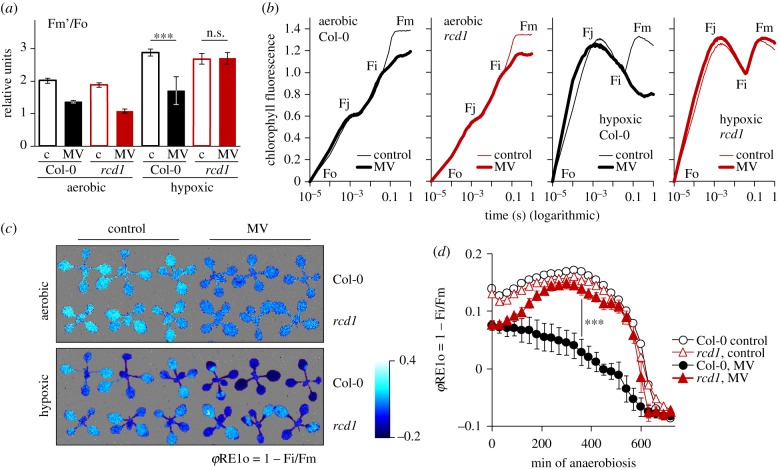
Hypoxic environment inhibits PSI–MV electron transfer in the *rcd1* mutant. Source data and statistical analyses are presented in electronic supplementary material, table S2. (*a*) Alterations in Fm’ induced by hypoxic conditions (20 min pre-treatment with nitrogen gas). Controls untreated with MV are labelled ‘c’. Mean values ± standard deviations are shown. ***P-value < 0.001, one-way ANOVA with Bonferroni corrected *post hoc* test. Full fluorescence kinetics is presented in electronic supplementary material, figure S3A. (*b*) OJIP kinetics of chlorophyll fluorescence recorded under aerobic or hypoxic conditions. Kinetics are double normalized to fluorescence at Fo and Fi (20 µs and 40 ms, accordingly). Of note is the increase of Fo–Fj phase observed in both lines in hypoxic conditions, which has previously been attributed to induced fermentative metabolism and over-reduction of the plastoquinone pool [50]. Similarities in hypoxia-induced changes of the Fo–Fj phase suggested that over-reduction of plastoquinone pool was similar in *rcd1* and Col-0. (*c*) False colour image of *φ*RE1o = 1 − Fi/Fm in plants treated as in (*b*). (*d*) Dynamics of *φ*RE1o in plants subjected to hypoxia in AnaeroGen. Mean values ± standard deviations are shown. ***P-value < 0.001, one-way ANOVA with Bonferroni corrected *post hoc* test (*N* = 2). Raw OJIP kinetics is presented in electronic supplementary material, figure S3D.

We thus exposed leaf discs to hypoxic atmosphere by flushing nitrogen gas for 20 min in darkness and monitored PET during 5 min of low intensity light as in figure 2*a*–*c*. In all the tested lines, hypoxia led to increased fluorescence, as compared to the aerobic controls (electronic supplementary material, figure S3A). This was anticipated, since O_2_ acts as an electron sink for several chloroplast processes including the Mehler reaction and activity of the chloroplast terminal oxidase PTOX. In Col-0, MV markedly diminished the hypoxia-related rise in chlorophyll fluorescence (figure 4*a*; electronic supplementary material, figure S3A). This was likely owing to the fact that MV catalysed the Mehler reaction, thus compensating for oxygen deficiency. Importantly, the same effect of MV was observed in the *ptox* mutant, indicating that it was not associated with the PTOX activity. Similarly, MV lowered chlorophyll fluorescence in hypoxia-treated *npq4* and *stn7*, suggesting that the shift was not owing to NPQ or chloroplast state transitions. In striking contrast to all of the above plant lines, in *rcd1*, MV did not lower chlorophyll fluorescence under hypoxic conditions (figure 4*a*; electronic supplementary material, figure S3A). This implies that in *rcd1*, but not in other lines, hypoxic environment compromised the electron flow through MV.

### (e) Hypoxia inhibits Photosystem I–methyl viologen electron transfer in *rcd1*, but not in Col-0

It remained unclear whether the hypoxic atmosphere affected MV activity directly at the electron-acceptor side of PSI, or indirectly, for example, through changes in mitochondrial respiration. To address this question, we imaged fast chlorophyll fluorescence rise induced by saturating light (OJIP, standing for Fo, Fj, Fi and Fp = Fm phases of fluorescence induction kinetics [51,52]) in dark-acclimated plants. The method made it possible to observe the changes in PET at much higher time resolution than the imaging PAM. In both Col-0 and *rcd1*, the activity of MV was visible already after 40 ms of illumination as lowered Fi–Fm phase of the OJIP kinetics (figure 4*b*). This effect has previously been ascribed to the oxidative action of MV on the electron-acceptor side of PSI [10,11,53]. It has been proposed that MV releases ‘a traffic jam of electrons caused by a transient block at the acceptor side of PSI’ [10], thereby lowering fluorescence. In hypoxic conditions, the effect of MV on the Fi–Fm phase was still detected in Col-0; however, it disappeared in *rcd1* (figure 4*b*). The Fi–Fm phase can be expressed by the parameter *φ*RE1o = 1 – Fi/Fm, called the quantum yield of the electron flux to PSI electron acceptors [52]. Accordingly, in aerobic conditions MV affected *φ*RE1o both in Col-0 and in *rcd1*, while in hypoxic conditions, the effect of MV was only observed in Col-0, but absent from *rcd1* (figure 4*c*). Importantly, in an analogous dark hypoxic assay *stn7* performed similarly to Col-0, while *rcd1 ntrc* was indistinguishable from *rcd1* (electronic supplementary material, figure S3B, C). This indicated that the differential response to MV was most probably not owing to dark anaerobic-induced state transitions [50] nor to NTRC-dependent CET. The disappearance of MV activity in *rcd1* as early as after 40 ms of illumination strongly indicated that hypoxia counteracted electron flow through MV directly at the electron-acceptor side of PSI, and not through the changes outside of the chloroplast.

Fast change in OJIP kinetics induced by nitrogen gas flush made it difficult to observe the transition of PET from the aerobic to the hypoxic state and to address the stability of the difference between the genotypes. Thus, we generated hypoxia with an alternative approach, using the AnaeroGen anaerobic gas generator, and imaged OJIP kinetics once in 30 min over 12 h of darkness. The raw OJIP kinetics are presented in electronic supplementary material figure S3D and the calculated *φ*RE1o parameter in figure 4*d*. The treatment resulted in similar changes of OJIP as those induced by nitrogen gas (figure 4*b*,*c*). In MV-treated *rcd1*, hypoxic atmosphere restored *φ*RE1o to the levels observed in MV-untreated controls, while this did not happen in MV-treated Col-0 (figure 4*d*). The difference between the genotypes remained stable during several hours of incubation, suggesting that it was not owing to possible alterations in gas exchange rates or different starting O_2_ reserves in Col-0 and *rcd1*. Collectively these experiments implied that hypoxic environment rapidly blocked the PSI–MV electron transfer in *rcd1*, but not in Col-0.

### (f) Increased expression of mitochondrial dysfunction stimulon genes is linked to hypoxic response

**Figure 5.**
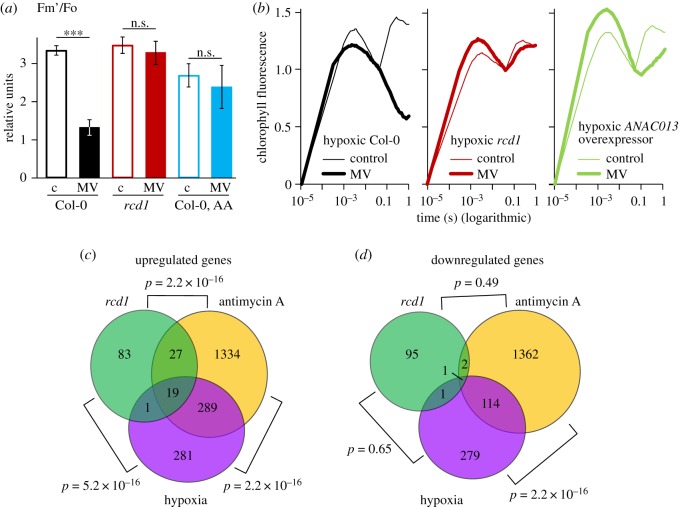
Expression of MDS genes is linked to hypoxic response. (*a*,*b*) Response to MV is sensitive to hypoxia in MDS-overexpressing plants other than *rcd1*. (*a*) The effect of hypoxia on electron transfer through MV in AA-treated Col-0. Fm’ was obtained as in (figure 4*a*). Controls untreated with MV are labelled ‘c’. Mean values ± standard deviations are shown. *** *P*-value < 0.001, one-way ANOVA with Bonferroni corrected *post hoc* test (*N* = 3). Full fluorescence kinetics is presented in electronic supplementary material, figure S4. (*b*) Similarly to *rcd1*, in *ANAC013* overexpressor line PSI–MV electron transfer was suppressed by hypoxia. The OJIP kinetics are double normalized to fluorescence at Fo and Fi (20 µs and 40 ms, accordingly) (*N* = 3). (*c*,*d*) Analysis of publicly available transcriptomic datasets obtained in the *rcd1* mutant, in Col-0 plants treated with AA or in Col-0 plants treated with hypoxia. Venn diagrams show the overlap of up- (*c*) and down-regulated (*d*) genes. Statistical analysis was performed by a pairwise Fisher's exact test.

The results indicated that the PSI–MV electron transfer was suppressed in *rcd1* during illumination or under hypoxic environment. The *rcd1* mutant is characterized by increased expression of the MDS genes, which results in altered mitochondrial respiration [3]. We thus assessed whether the altered response to MV under hypoxia observed in *rcd1* exists in other MDS-inducing lines or treatments. The mitochondrial electron transfer inhibitor antimycin A (AA) activates MDS retrograde signalling. Accordingly, in wild-type plants pre-treated with AA, hypoxia led to decreased electron transfer through MV (figure 5*a*; electronic supplementary material, figure S4). Measurement of OJIP kinetics in plants overexpressing *ANAC013* [1,2] revealed the suppression of PSI–MV electron transfer by hypoxia similar to that in *rcd1* (figure 5*b*). These observations demonstrated that oxygen availability affected the MV response not only in *rcd1*, but also under other perturbations activating MDS gene expression. Thus, the studied effects are not a peculiarity of *rcd1*, but rather a common feature associated with MDS overexpression.

Mitochondrial AOXs were proposed to be implicated in MV tolerance [3]. To test the contribution of the individual AOX isoforms, we performed analogous experiments in *aox1a*, *aox1c* and *aox1d* knockout mutants and in the *AOX1a* overexpressor line [30]. All of them showed wild-type response to hypoxia (electronic supplementary material, figure S5A). Similarly, the *rcd1 aox1a* double mutant [31] was indistinguishable from *rcd1* (electronic supplementary material, figure S5B). Thus, the studied effect on chloroplast electron transfer could not be attributed to a single AOX isoform.

Taken together, the results demonstrated altered response to the hypoxia of the MDS-overexpressing plants. Interestingly, transcriptomic changes occurring in plants exposed to hypoxia are similar to the changes triggered by AA [54]. Thus, the two types of stress are also linked in physiological conditions unrelated to MV. To find out whether similar changes in gene expression occurred in *rcd1*, we compared publicly available transcriptomic datasets obtained in the *rcd1* mutant and in Col-0 treated with AA or hypoxia. The lists of genes with changed expression under these three perturbations showed a statistically significant overlap (figure 5*c*,*d*). The 19 genes that were activated in all the perturbations included the hypoxia-responsive universal stress protein 1 (*HRU1*), the stress-responsive transcription factor *ZAT10*, transcription factor *WRKY25* and the MDS genes *AOX1a* and *SOT12*. These results implied that transcriptional reprogramming induced by hypoxia bears similarity to the changes in gene expression triggered by MDS signalling and to those observed in *rcd1* under standard growth conditions (figure 5*c*,*d*; electronic supplementary material, table S3).

## 4. Discussion

Expression of the MDS genes is related to mitochondrial retrograde signalling, but it also affects chloroplast functions. One prominent feature of MDS-overexpressing plants is their tolerance to MV [1–3]. The Arabidopsis mutant *rcd1* that among several distinct phenotypes [55] overexpresses MDS genes and is MV tolerant [3,31] provides an experimental tool to dissect the interaction between the organelles. MV tolerance of *rcd1* was suggested to be owing to altered redox state of chloroplast thiol enzymes, and/or modified electron transfer pathways downstream of PSI [3]. Knockout of the master regulatory chloroplast thiol enzyme NTRC in the *rcd1* background largely suppressed *rcd1* phenotypes related to PET (figures 1 and 3). However, the long-term tolerance of *rcd1 ntrc* to MV-induced PSII inhibition was only slightly lower than in *rcd1* (figure 1*d*). These observations imply that the more reduced states of thiol enzymes are not the primary reason for MV tolerance of *rcd1*.

Several recent studies have suggested that ROS generated by the Mehler reaction are the main electron sink for chloroplast 2-CP and its reductants, including NTRC [19–21]. Thus, *rcd1*-specific alternations of the Mehler reaction may underlie both its MV tolerance and more reduced state of 2-CP [3] and NTRC (figure 1). Consequently, the changes in the activities of NTRC and its targets affect PET, including operation of the NDH complex (figure 1) and possibly of the malate shuttle [3]. Thus, we suggest that the following chain of events could be responsible for the control of chloroplast processes by MDS gene products. The signals initiated in the mitochondria induce expression of MDS genes. This affects electron transfer at the electron-acceptor side of PSI suppressing the Mehler reaction and ROS production in the chloroplast. Lower ROS production leads to more reduced chloroplast thiol enzymes, which alters photosynthesis.

The question of how MDS gene products influence the chloroplast Mehler reaction remained unanswered. In search for mediators linking the two organelles, we discovered that hypoxic treatment in darkness caused rapid inactivation of PSI–MV electron transfer in *rcd1*, but not in Col-0 (figure 4). One possible explanation for this is that in *rcd1* unknown readjustments of PET disrupt the access of MV to PSI. Such readjustments would likely be post-translational, as the change occurred during only 20 min of hypoxic treatment. The changes were not associated with dark hypoxia-induced state transitions [50] because the *stn7* mutant, deficient in state transitions, performed in a similar way to Col-0 that had functional state transitions [50] (electronic supplementary material, figure S3B). The effect was also not owing to the NTRC-dependent CET, as *rcd1 ntrc* performed similarly to *rcd1* (electronic supplementary material, figure S3C). Further research into chloroplast electron flows in *rcd1* and, more generally, in MDS-overexpressing plants, is required to address the implication of other possible CET pathways in MV tolerance.

As another possibility, the *rcd1*-specific response could rely on some other pre-existing adaptations. Several comprehensive studies in *rcd1* did not reveal chloroplast defects that could unambiguously explain its MV tolerance [3,6,7]. By contrast, defects have been found in the *rcd1* mitochondria. The mutant has increased respiration metabolic flux (electronic supplementary material, table S1) and higher AOX capacity [3]. AOX activity is implicated in the control of PET [3,22–25] and in tolerance to MV [3]. Thus, AOXs and/or other mitochondrial components could be involved in the *rcd1*-specific reaction to the hypoxic treatment in darkness.

AOX respiration consumes reducing power and molecular oxygen [22]. Hence, one possible mechanism connecting AOX activity to the Mehler reaction is electron transfer between the organelles. Photorespiration [26] and malate shuttle [3] have been proposed to mediate this pathway. Importantly, however, the hypoxic treatments described in figure 4*b*–*d* were performed in darkness, ruling out the involvement of photorespiration, NADPH-MDH-dependent malate shuttle and NADP-GAPDH-dependent glyceraldehyde 3-phosphate shuttle, as these pathways are light dependent [56].

Alternatively, AOXs and/or other MDS components could affect the response to MV through decreasing cellular availability of O_2_. AOXs have previously been proposed to act as oxygen sink in plant mitochondria [57,58] and non-photosynthetic tissues [59,60]. In photosynthesizing plants, this effect is certainly masked by O_2_ evolution. However, the results described in figure 4*b*–*d* were obtained in dark-acclimated plants using a 1 s flash of saturating light. Hence, photosynthetic O_2_ evolution was inactive. In darkness, increased respiratory activity possibly makes *rcd1* plants more dependent on the uptake of atmospheric O_2_ through stomata. Therefore, disrupting external O_2_ supply could lead to higher O_2_ deficit in *rcd1* compared to Col-0. This could explain the suppressed PSI–MV electron transfer in *rcd1* (figure 4).

It remains unclear how the effects observed in *rcd1* in darkness under hypoxic conditions are related to the mutant's long-term MV tolerance (e.g. figure 1*d*), or to specific light-dependent alterations of PET (figure 3), occurring in *rcd1* under aerobic atmosphere. It is worth pointing out that according to our results the treatment with MV in light rapidly suppressed photosynthetic O_2_ evolution in both *rcd1* and Col-0, while respiration remained unaffected (figure 2 and electronic supplementary material, figure S1). Besides, MV has been shown to promote stomatal closure [61], which could limit the supply of atmospheric O_2_. This hints that the treatment of plants with MV can be associated with O_2_ deficiency in photosynthesizing tissues. However, the direct assessments of *in vivo* O_2_ tissue concentrations are needed to test this assumption. Transcriptomic meta-analysis revealed similarities of gene expression changes induced by AA, in the *rcd1* mutant and by hypoxia (figure 5; electronic supplementary material, table S3). This suggests that the expression of MDS genes is linked to hypoxic response not only in artificial physiological situations such as MV treatment, but also in standard growth conditions. Whether this similarity is related to altered cellular O_2_ availability is yet to be determined. Our observations open up new experimental possibilities to explore the mechanisms of interactions between the mitochondria and the chloroplasts.

## Supplementary Material

Supplementary figures

## Supplementary Material

Supplementary Table S1. Metabolic analyses

## Supplementary Material

Supplementary table S2. Source data and statistics

## Supplementary Material

Supplementary table S3. Genes induced in rcd1, AA, hypoxia

## Data accessibility

Data are presented as electronic supplementary material.
