# Supplementary material for: Dissecting the interaction of photosynthetic electron transfer with mitochondrial signalling and hypoxic response in the Arabidopsis rcd1 mutant
Source: Philos Trans R Soc Lond B Biol Sci. 2020 May 4;375(1801):20190413. doi: 10.1098/rstb.2019.0413 (PMC7209945; doi:10.1098/rstb.2019.0413)
Supplement: Supplementary figures [file rstb20190413supp1.pdf]

PHILOSOPHICAL TRANSACTIONS B
royalsocietypublishing.org/journal/rstb

Dissecting the interaction of photosynthetic electron transfer with mitochondrial signalling and hypoxic response in the Arabidopsis *rcd1* mutant

Alexey Shapiguzov, Lauri Nikkanen, Duncan Fitzpatrick, Julia P. Vainonen, Richard Gossens, Saleh Alseekh, Fayezeh Aarabi, Arjun Tiwari, Olga Blokhina, Klára Panzarová, Zuzana Benedikty, Esa Tyystjärvi, Alisdair R. Fernie, Martin Trtílek, Eva-Mari Aro, Eevi Rintamäki, Jaakko Kangasjärvi.

Supplementary figures S1-S5

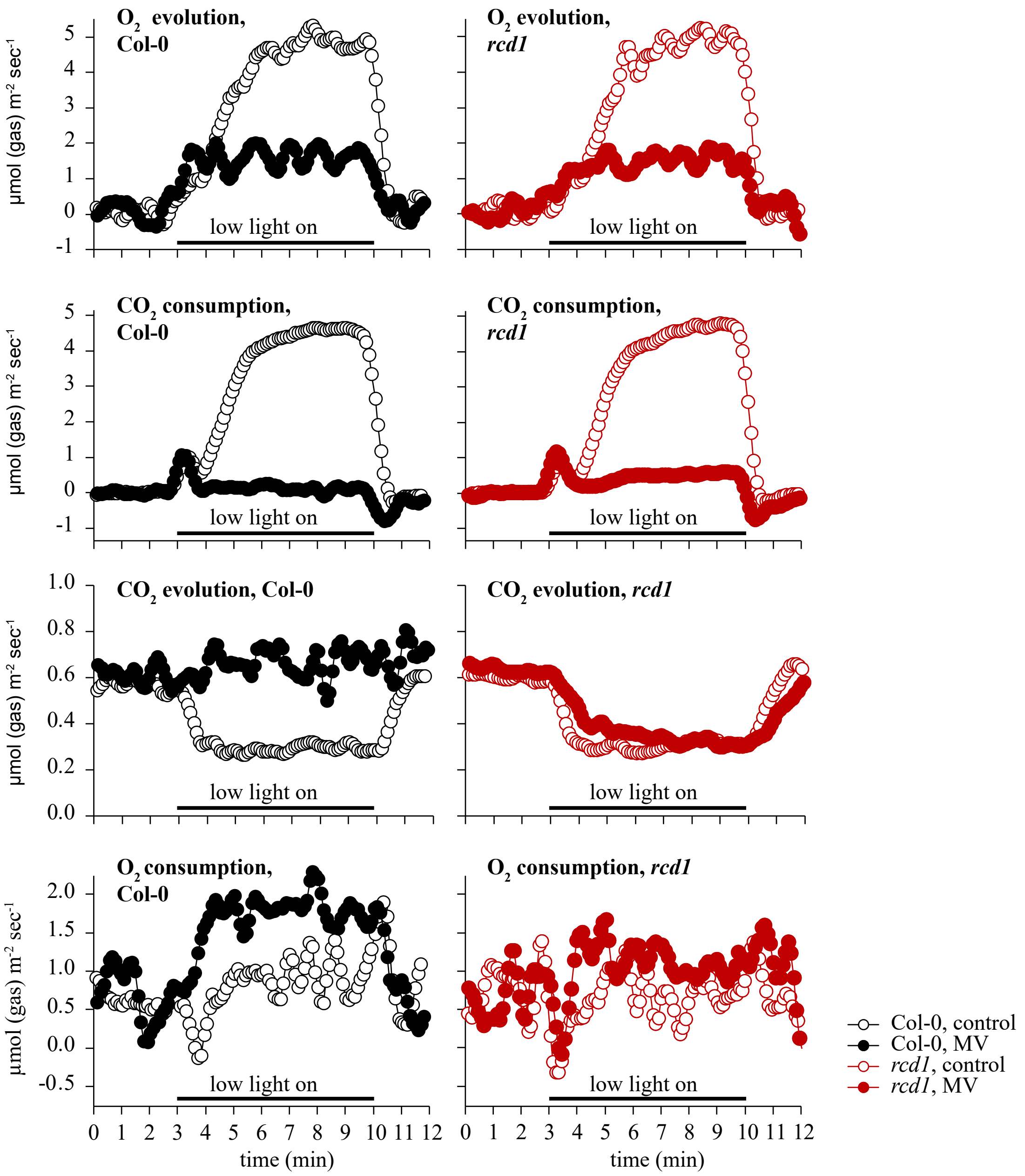

Supplementary figure S1. Gas exchange in Col-0 and *rcd1* leaf discs treated or untreated with MV, as monitored by MIMS. The kinetics of O_2 evolution in Col-0 is presented in the main figure 2*g*. O_2 evolution in *rcd1* is indistinguishable from the wild type. Kinetics of CO_2 evolution can be used to estimate the rate of mitochondrial respiration. Treatment with MV did not affect dark CO_2 evolution either in Col-0 (left) or in *rcd1* (right). When plants were exposed to light, photosynthetic carbon fixation reabsorbed a fraction of CO_2 produced by respiration, thus lowering net CO_2 emission [1]. Treatment with MV prevented light-dependent CO_2 reabsorption in Col-0, but not in *rcd1*. This suggested that both photosynthesis and respiration were active in MV-treated *rcd1*, while only respiration was active in MV-treated Col-0.

[1] Busch FA, Sage TL, Cousins AB, Sage RF. 2013 C_3 plants enhance rates of photosynthesis by reassimilating photorespired and respired CO_2. *Plant Cell Environ*, **36**, 200-212. (doi:10.1111/j.1365-3040.2012.02567.x)

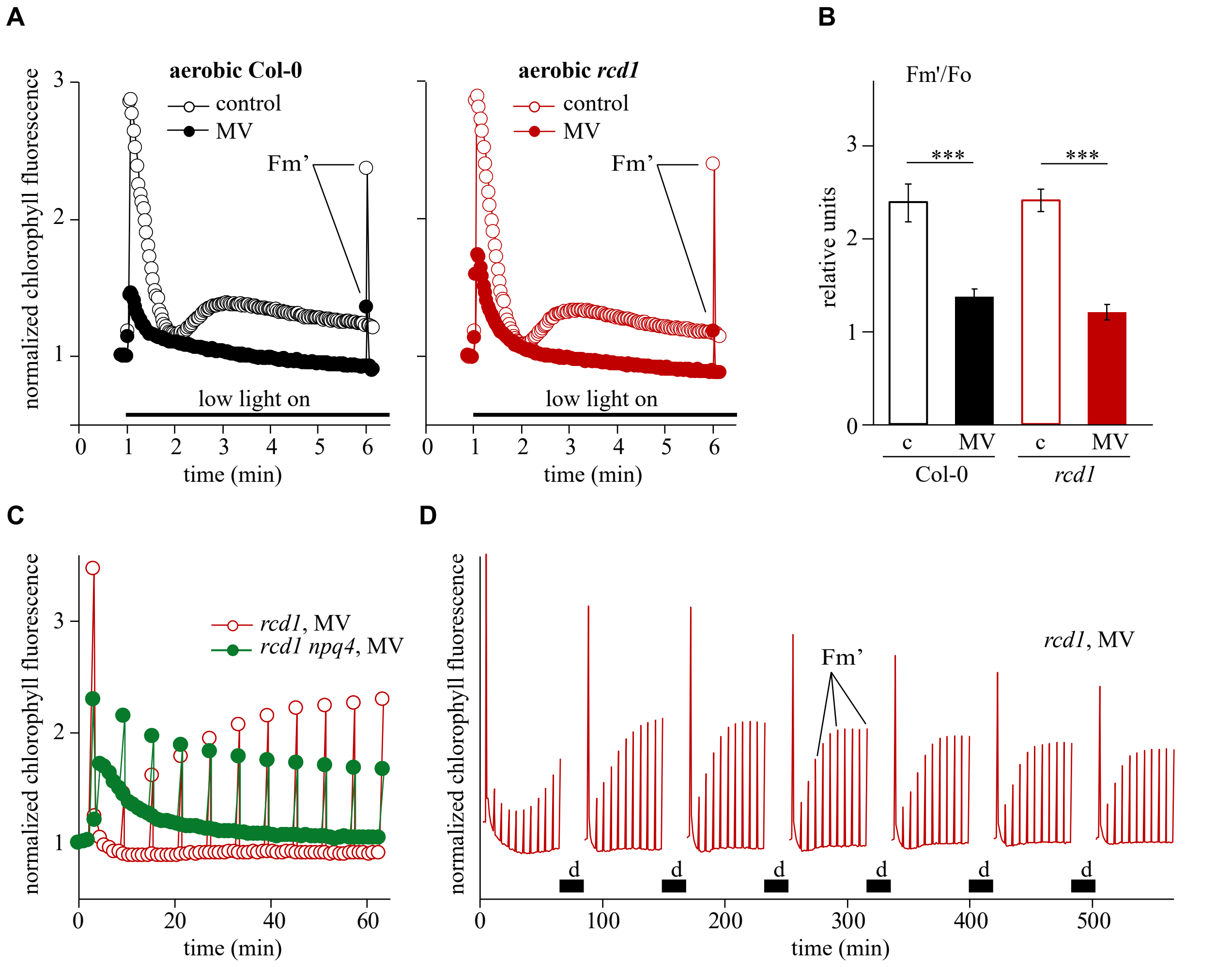

Supplementary figure S2. The effects of MV on chlorophyll fluorescence during exposure to low light. (A) Kinetics of chlorophyll fluorescence in Col-0 and in *rcd1* mutant measured as in the main figure 2*a*,*b*. The reads are normalized to Fo. (B) Quantification of Fm' parameter measured in (A). Mean values ± standard deviations are shown. *** P value < 0.001, one-way ANOVA with Bonferroni corrected post hoc test. (C) Long illumination led to recovery of Fm' in MV-treated *rcd1*, but not in MV-treated *rcd1 npq4*. The reads are normalized to Fo. (D) The response of *rcd1* to MV is "reset" in darkness. Introducing dark periods ("d") in the course of light exposure temporarily restored NPQ (as seen by lowered Fm') in MV-treated *rcd1*. Thus, light-dependent inhibition of physiological activity of MV in *rcd1* was reversible by darkness.

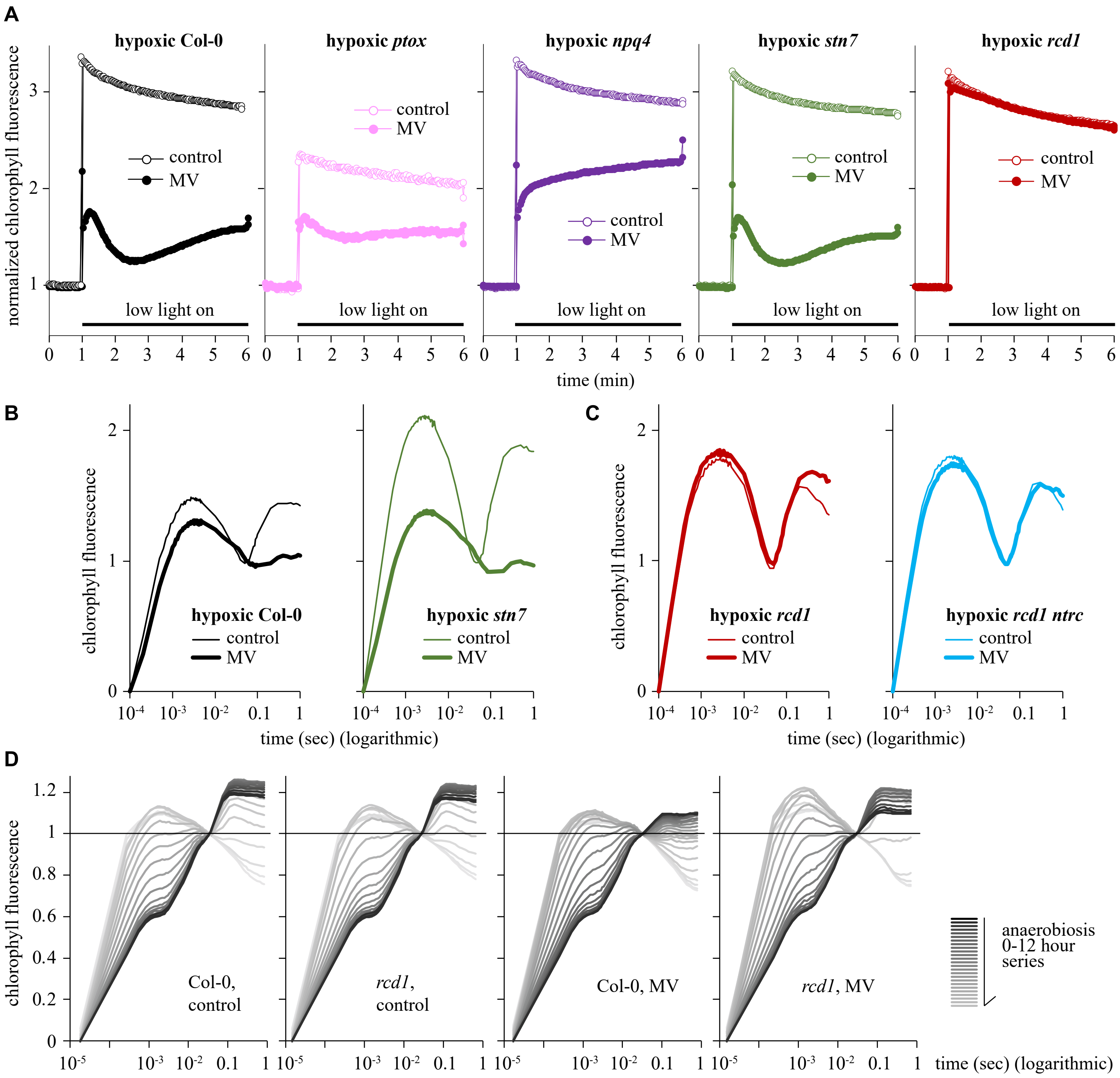

Supplementary figure S3. Measurements of chlorophyll fluorescence in hypoxic atmosphere.
(A) Chlorophyll fluorescence of leaf discs was recorded after 20-min treatment with nitrogen gas in darkness. In all the tested lines except *rcd1*, leaf discs pre-treated with MV demonstrated quenched chlorophyll fluorescence as compared to MV-untreated controls. The presence of this effect in *ptox*, *npq4*, and *stn7* mutants suggested that it was not mediated by PTOX chloroplast terminal oxidase, NPQ, and chloroplast state transitions, accordingly. The reads are normalized to Fo obtained in dark-adapted hypoxic conditions. (B) OJIP kinetics was recorded using 1-second flash of saturating light in dark-acclimated plants that were pre-treated with hypoxic atmosphere for 20 min. The response of the *stn7* mutant deficient in state transitions was similar to wild-type. (C) The *rcd1 ntrc* mutant with compromised CET was indistinguishable from *rcd1*. (D) Dynamic response of OJIP kinetics in MV-treated Col-0 and *rcd1* leaf discs subjected to hypoxia in AnaeroGen (N = 2). The extracted parameter φRE1o = 1 – Fi/Fm is presented in the main figure 4*d*. The OJIP kinetics are double normalized to fluorescence at Fo and Fi (20 µsec and 40 msec, accordingly).

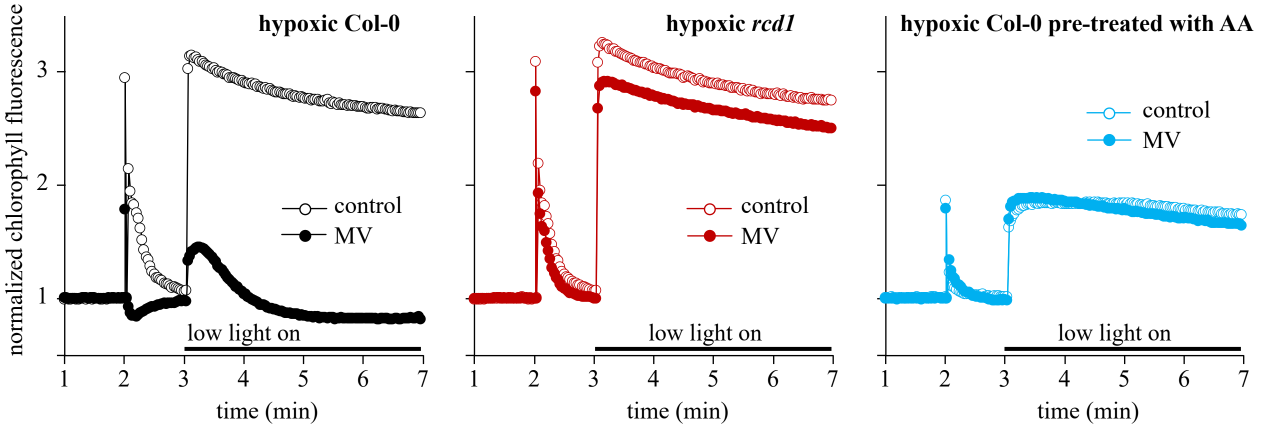

Supplementary figure S4. Response to MV is sensitive to hypoxia in plants treated with antimycin A. Pre-treatment of wild-type plants with 2.5 µM antimycin A (AA) followed by 20-min hypoxic treatment abolished the effect of MV on PET. Chlorophyll fluorescence kinetics was normalized to Fo obtained under dark-adapted hypoxic conditions. Quantification of Fm' is presented in the main figure 5*a* (N = 3).

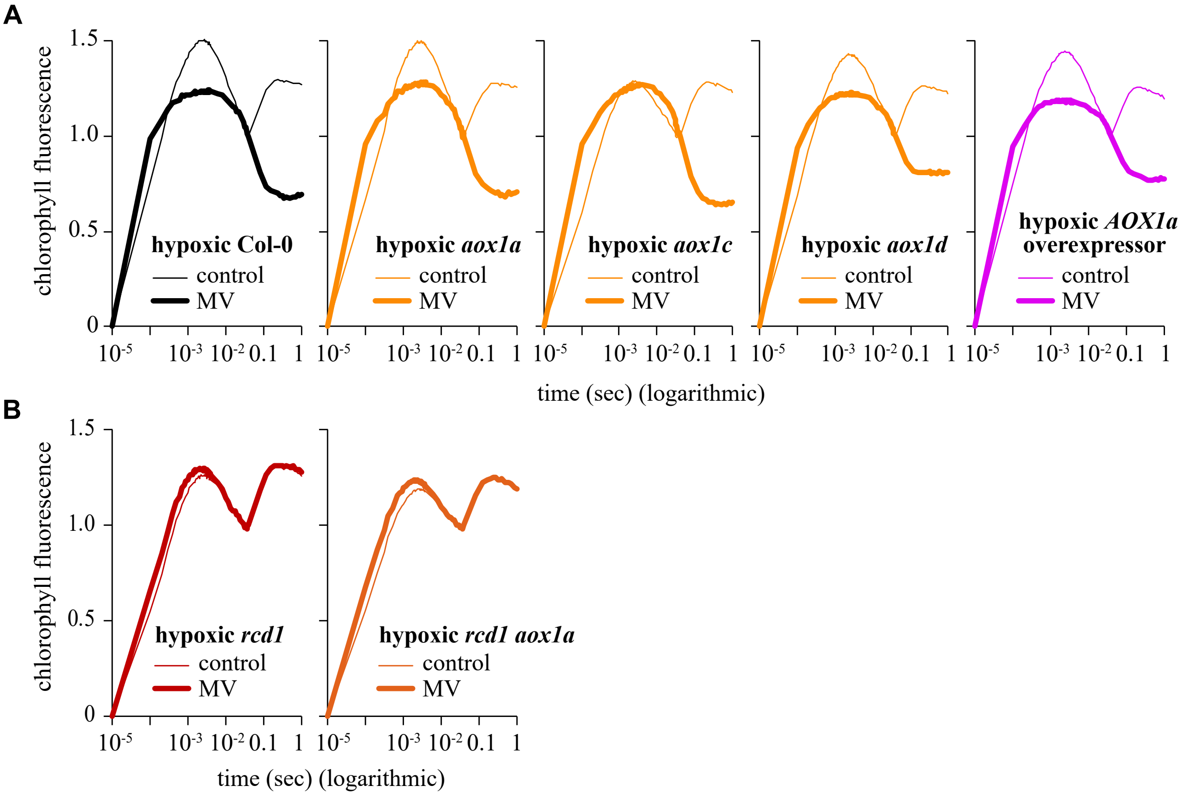

Supplementary figure S5. Response to MV in the mutants deficient in AOX isoforms. (A) In the *aox1a*, *aox1c* and *aox1d* mutants deficient in AOX isoforms, and in the line overexpressing *AOX1a*, chlorophyll fluorescence under hypoxia was sensitive to MV similarly to Col-0. (B) In the *rcd1 aox1a* double mutant chlorophyll fluorescence under hypoxia was insensitive to MV similarly to *rcd1*. The OJIP kinetics are double normalized to fluorescence at Fo and Fi (20 µsec and 40 msec, accordingly) (N = 2).
